# Prenatal Mental Representations in Italian First-Time Mothers Before and During the COVID-19 Pandemic: A Study with Interviews on Maternal Representations During Pregnancy

**DOI:** 10.1007/s10995-022-03573-5

**Published:** 2023-01-31

**Authors:** Martina Smorti, Giulia Mauri, Alessia Carducci, Angelica Andreol, Lucia Bonassi

**Affiliations:** 1 Department of Surgical, Medical and Molecular Pathology and Critical Care Medicine, University of Pisa, Via Savi 10, 56126 Pisa, Italy; 2 Department of Mental Health, ASST Bergamo-Est, Seriate, Italy

**Keywords:** Primiparous women, Maternal representation style, COVID-19, Content analysis, Interview on maternal representations during pregnancy

## Abstract

**Introduction:**

Psychosocial risks increase the levels of not-integrated/ambivalent and restricted/disengaged representations during pregnancy, but no study has specifically analysed the impact of the COVID-19 pandemic on maternal representation styles.

**Objectives:**

(1) to compare maternal representation styles in primiparous women who became pregnant before and during the COVID-19 pandemic and (2) to analyse the content of representation styles during the COVID-19 pandemic.

**Methods:**

A total of 37 Italian pregnant women were recruited from 2019 to 2021. The sample was divided into two groups: the pre-COVID-19 group (22 women, mean age = 33.14 years; SD = 3.78) and the COVID-19 group (15 women, mean age = 35.9 years; SD = 4.6). Interviews on maternal representations during pregnancy were administered and analysed for style and content. Results: Women during the COVID-19 pandemic reported more restricted/disengaged and less integrated/balanced representation styles than women pre-COVID-19. Content analysis showed that the COVID-19 pandemic led women to focus more on concrete aspects of pregnancy in lieu of emotional aspects, thus leading them to develop more restricted/disengaged representation styles.

**Conclusions for practice:**

In future pandemics pregnant women should be supported in focusing their attention to emotions, sensations and fantasies about themselves as mothers and their children.

**Supplementary Information:**

The online version contains supplementary material available at 10.1007/s10995-022-03573-5.

## Introduction

During the pregnancy of the first-child, a woman’s identity is modified through the restructuring of mental self-representation and the elaboration of relationships with significant others (Slade et al., 2009; Stern, 1995). Mental self-representations are enriched with the maternal component; the representation of the couple progressively integrates the family image, and the marital relationship is improved with the parental component (Cast, 2004).

Ammaniti and colleagues (1995), using the Interview on maternal representations during pregnancy (Intervista sulle Rappresentazioni Materne in Gravidanza – IRMAG), identified seven specific dimensions of maternal narratives that allow to identify three representation styles related to how a woman deals with motherhood and the relationship with her unborn child: the integrated/balanced, restricted/disengaged, and not-integrated/ambivalent. Integrated/balanced representation styles are typical of women who establish a relationship with their unborn child, developing a real dialogue with him or her from the beginning of pregnancy. Women with integrated representation styles tend to be open to changes during pregnancy and to experience intense emotional involvement related to motherhood. Restricted/disengaged representation styles are typical of women who rationalize the experience of motherhood and do not become involved in the experience of pregnancy or in a relationship with their unborn child. Not-integrated/ambivalent representation styles are typical of women who have ambivalent feelings towards motherhood that oscillate between excessive involvement and struggles to distance themselves (Ammaniti et al., 1995).

Representation styles during pregnancy could influence the relationship between a woman and her baby (Ammaniti et al., 2007; Stern et al. 1995; Tambelli et al., 2008). Specifically, when a woman has a representation style that is open to change and flexible, she can “have an unconditional acceptance of the infant and a realistic consideration of the baby’s individual characteristics and of any difficulties emerging in the relationship with him or her” (Tambelli et al., 2014, pp. 378–379).

However, psychosocial or depressive risk factors may interfere with the development of mental representation styles, negatively affecting a woman’s interaction with her child (Ammaniti et al., 2013; Ammaniti & Speranza, 1995; van Bussel et al., 2009).

In particular, depressed women tend to report restricted representations during pregnancy and to show a defect in reciprocal regulation with the baby and poor care behaviours after birth (Ammaniti et al., 2007). Moreover, when pregnant women experience psychosocial risks (such as poor social support, socioeconomic stress, and conflict in marital or parental relationships) in addition to depressive symptoms, they tend to develop not-integrated representations and to report poor emotional exchanges with their baby (Agostini et al., 2009; Ammaniti et al., 2013; Smorti et al., 2010). In summary, the quality of mental representations during pregnancy could be compromised by specific risk conditions (i.e., depressive and psychosocial risks) that, through the development of not-integrated and restricted representations, have a negative impact on maternal behaviours (Agostini et al., 2009; 2013; Davis-Floyd et al., 2020; Pajulo et al., 2001; Smorti et al., 2010).

The COVID-19 pandemic and consequent restrictions increased social isolation (Brooks et al., 2020), insecurities, and difficulties in accessing one’s support systems (Diamond et al., 2020), enhancing the levels of depression in mothers-to-be compared to those who became mothers before the pandemic (Lebel et al., 2020; Ostacoli et al., 2020; Smorti et al., 2021; 2022; Zanardo et al., 2020). Thus, the COVID-19 pandemic and related restrictions represent a cumulative risk factor (psychosocial and depressive) (Diamond et al., 2020) for a difficult transition to motherhood (Hassanzadeh et al., 2021).

National and health care restrictions aimed at protecting physical health for mothers and babies have modified traditional prenatal care (Diamond et al., 2020). The change of in-person obstetric visits to only allowing women to attend without the presence of a source of support (such as their partner) (Wilson et al. 2021) has changed the pregnancy-related rituals that constitute a space-time context to create meaning around the transition to motherhood (Imber-Black, 2002). At the same time, the higher attention to physical security in lieu of emotional health by hospital settings and health care staff (Delmastro & Zamariola, 2020) may have led women to devote more attention to the concrete aspects of pregnancy in lieu of the emotional aspects during the COVID-19 pandemic.

Despite the relevance of this issue, two years after the outbreak of COVID-19, little attention has been given to the impact of the COVID-19 pandemic on the style and content of mental representations. This aspect seems to be particularly relevant for first-time mothers in the Italian context due to Italy having the most severe and longest restrictive measures in Europe in 2020 (BBC https://www.bbc.com/news/av/world-europe-52400085).

### Objectives

Starting from these considerations, this research had the following aims: (1) to compare the seven dimensions of maternal representations reported by primiparous women who experienced pregnancy in the pre-COVID-19 period (pre-COVID-19 group) with those reported by primiparous women during the COVID-19 pandemic (COVID-19 group); (2) to compare the maternal representation styles expressed by primiparous women in the pre-COVID-19 and COVID-19 groups; and (3) to explore the content of maternal representations (Ammaniti et al., 1995, 2013) expressed by primiparous women in the COVID-19 group. In line with a previous study (Tambelli et al., 2014), we hypothesized that the COVID-19 group (due to multiple risk factors) would present lower level of integrated/balanced representations and higher level of restricted/disengaged and not-integrated/ambivalent representations than the pre-COVID-19 group.

## Methods

### Participants and Procedure

This study was part of a larger multicentre research project on perinatal well-being. According to the study design and the aims of the research, a sample of Italian primiparous pregnant women was recruited from January 2019 to May 2021 from two participating institutions in northern and central Italy. This study followed the COREQ criteria for reporting qualitative research. The study was conducted in accordance with the ethical standards laid down in the 1964 Declaration of Helsinki and its later amendments. After the Ethical Committee of the Local Health Authorities approved the study, women in the waiting room of the hospital’s obstetric unit before their routine third-trimester medical visit were invited to participate. The inclusion criteria were as follows: (a) women aged > 18 years old; (b) women who were able to speak and read Italian; (c) women with low-risk pregnancies; and (d) primiparous women. The exclusion criteria were as follows: (a) women with a psychiatric diagnosis; (b) women with pregnancy medical complications; and (c) women with the diagnosis of foetal genetic conditions or congenital abnormalities. The inclusion and exclusion criteria were defined and agreed upon with clinicians. A female researcher (Psy.D.) was responsible for the informed consent process, highlighting the study’s purpose and aims. The researcher explained that participation included an in-person interview that should be arranged on the hospital premises. All participants were informed that the interview would be recorded and that they should give written permission to record the interview. Participation was voluntary, with no monetary incentive and with the possibility of withdraw from the study at any time. All persons gave their informed consent prior to their inclusion in the study. Details that might disclose the identity of the subjects under study have been omitted.

A few weeks after their recruitment, individuals were contacted by phone by the researcher to arrange an in-person interview on the hospital premises. An experienced female researcher (Psy.D. with over ten years of experience with the IRMAG) conducted the interview, which lasted approximately 45 min. No one other than the participant and researcher was present during the interview. All interviews were audio-recorded and transcribed verbatim. All audio recordings and their transcripts were uploaded to a password-protected folder for each hospital to which only the researchers involved had access. Each recording had a unique identification code to preserve the anonymity of participants. Only individuals who completed the entire procedure were included in the study.

Twenty-four consecutive participants who met the inclusion criteria were contacted for this study during the COVID-19 pandemic (from 3/2020 to 5/2021); three (12.5%) refused to participate due to a lack of time or the fear of contagion, and six (25%) gave their consent but were unable to participate in the in-person interview due to personal COVID-19 infection (n = 4) and/or quarantine measures (n = 2). Consequently, only fifteen participants (mean age = 35.9 years; SD = 4.6) who completed the procedure were included in the study during the pandemic (mean gestational weeks = 31.40; SD = 1.40) (COVID-19 group).

To create a comparison group, a sample was drawn from a larger population recruited for the research project in the prepandemic period (from 1/2019 to 2/2020) and randomly selected, taking the sociodemographic characteristics of the COVID-19 group into account, such as the mean age and mean gestational weeks. Therefore, a total of twenty-two pregnant women (mean age = 33.14 years; SD = 3.78) were included as the pre-COVID-19 group (mean gestational weeks = 31.14; SD = 1.32).

### Measures

Participants were asked to complete a questionnaire to obtain sociodemographic (nationality, age, education level, work status and marital status) and clinical data (weeks of gestation, spontaneous conception or medically assisted procreation, previous miscarriage).

To explore the mental representations, the Interview on maternal representations during pregnancy (IRMAG) was administered between the 28th and 32nd weeks of gestation (Ammaniti et al., 1995), in line with previous studies (Agostini et al., 2009; Ammaniti et al., 2007, 2013; Smorti et al., 2010). The IRMAG is a 41-question semistructured interview exploring six specific areas: (1) the desire for motherhood in the individual’s and couple’s history; (2) emotional reactions to pregnancy (personal, couple and family emotions); (3) emotions and changes during pregnancy in the woman’s and couple’s life and in family relationships; (4) the woman’s perceptions, emotions and fantasies about the “internal child”; (5) the woman’s future expectations about herself as a mother and her child; and (6) the woman’s biographical perspective regarding her present and past role as a daughter.

The IRMAG investigates the way in which the woman organizes, within a narrative structure, her experience of motherhood, in the representation of herself as a mother and in the representation of her child. Narratives are coded on a 5-point Likert scale (1 = poor; 5 = very marked) as a function of seven dimensions:

*Richness of perceptions* refers to the lack or richness of a mother’s perceptions about herself and the baby;

*Openness to change* refers to a mother’s ability to integrate new information about herself/the baby during pregnancy;

*Intensity of investment* refers to the extent to which a woman expresses affective engagement in describing both herself as a mother and her child;

*Coherence* refers to the organization of thoughts and feelings in a woman’s representation of herself and her child;

*Differentiation* refers to similarities or differences between a woman’s characteristics and her mother’s, partner’s, and family’s characteristics and the differentiation between herself and her baby;

*Social dependence* refers to the dependence of a woman’s representation of herself and her baby on the judgements, ideas and attitudes of others;

*Dominance of fantasies* refers to a mother’s coloured or distorted fantasies about herself as a mother and her child.

These dimensions allow to classify the interviews into one of three categories: integrated/balanced, restricted/disengaged and not-integrated/ambivalent (Ammaniti et al., 1995).

Two trained female professionals (Psy.D. with over ten years of experience with the IRMAG) independently coded the interview transcripts for classifications of dimensions and style (Ammaniti et al., 1995). The degree of interrater reliability ranged from 0.85 for richness of perceptions to 0.96 for coherence.

Moreover, content analysis of the interview transcripts was performed to explore the main themes concerning the impact of the COVID-19 pandemic on the experience of pregnancy and transition to motherhood.

Qualitative content analysis was performed for each of six interview areas by two independent female coders (Psy.D. with over three years of experience). This analysis intended to deepen the pregnancy experience (both internal and external) during the pandemic period. Since the purpose of this study was to evaluate the weight of the COVID-19 pandemic on pregnant women, it was necessary to analyse the contents of the experience as narrated by women. The themes were identified in advance in reference to the hypothesis about the impact of the COVID-19 pandemic and later refined based on reading the interview. Details about the qualitative content analysis are reported in Supplemental Material 1. The two coders read and reread the transcripts to identify themes, salient points, and deviations from the trends (Bowling, 2014). After reading the transcripts, the two coders jointly classified the content, working to come to an agreement, and in cases of disagreement, a third coder was involved.

### Data Analysis

Quantitative data were analysed using SPSS version 24. Descriptive statistics of quantitative data were performed for all dimensions. Chi-square analyses and t tests for independent samples were carried out to examine whether the groups (pre-COVID-19/COVID-19) differed in demographic and clinical variables, representation subscales, and maternal representation styles.

The alpha level was set to p = .05 for all tests with a confidence interval of 95%.

## Results

The sample consisted of 37 pregnant women divided into two groups. The demographic and obstetric data of the two groups are reported in Table 1.

**Table 1:** Demographic, obstetric and clinical characteristics of sample

	Pre-Covid (n = 22)	Covid (n = 15)	Statistics	p
*Age, Mean (SD)*	33.1 (3.7)	35.9 (4.6)	t_(35)_=-2.00	0.053
*Educational level, n (%)*			χ^2^_(4)_ = 1.68	0.80
Secondary school	2 (9.1%)	1 (6.7%)		
High-School	11 (50.0%)	5 (33.3%)		
First level Degree	3 (13.6%)	2 (13.3%)		
Master Degree	4 (18.2%)	5 (33.3%)		
Post-Degree specialization/ PhD	2 (9.1%)	2 (13.3%)		
*Employment status, n (%)*			χ^2^_(2)_ = 2.18	0.33
Precarious	3 (13.6%)	4 (26.7%)		
Housewife	2 (9.1%)	0		
Permanent	17 (77.3%)	11 (73.3%)		
*Employment status during pregnacy, n (%)*			χ^2^_(3)_ = 5.72	0.12
Maternity	15 (68.2%)	7 (46.7%)		
Dismissal	2 (9.1%)	1 (6.7%)		
No changes	3 (13.6%)	7 (46.7%)		
Other	2(9.1%)	0		
*Marital Status, n (%)*			χ^2^_(1)_ = 2.22	0.13
Living apart together	3 (13.6%)	0		
Married/ Cohabiting	19 (86.4%)	15 (100%)		
*Gestational weeks, Mean (SD)*	31.14 (1.3)	31.40 (1.4)	t_(35)_=-0.57	0.57
*Previous Miscarriage n (%)*			χ^2^_(1)_ = 0.37	0.53
No	18 (81.8%)	11 (11.7%)		
Yes	4 (18.2%)	4 (26.7%)		

No significant differences were found between the pre-COVID-19 and COVID-19 groups with respect to mean age, mean gestational age, education level, employment status, marital status, previous miscarriage, or employment status during pregnancy.

### Maternal Representation Dimensions and Style in The Two Groups

Table 2 describes the dimensions and styles, derived by the IRMAG, reported in the pre-COVID and COVID-19 groups.

**Table 2:** Descriptive of Maternal Representation Dimensions and Maternal Representation Style reported in pre-COVID and COVID group. Differences among groups are reported as result of statistical analysis

	Pre-Covid (n = 22)	Covid (n = 15)	Statistics	p
*Maternal representation Dimensions, Mean (SD)*				
Richness of perceptions	3.27 (0.9)	2.53 (0.6)	t_(35)=_2.66	0.012
Openness to change	3.27 (0.9)	2.46 (0.7)	t_(35)=_2.68	0.011
Intensity of investment	3.40 (0.6)	2.40 (0.6)	t_(35)=_4.96	0.000
Coherence	3.45 (0.9)	2.33 (0.8)	t_(35)=_3.82	0.001
Differentiation	3.09(0.8)	2.33 (0.6)	t_(35)=_3.23	0.003
Social dependence	2.36 (0.5)	2.60 (0.6)	t_(35)=_-1.27	0.21
Dominance of fantasies	2.63 (0.6)	2.26 (0.6)	t_(35)=_1.73	0.09
*Maternal representation Style, n (%)*			χ^2^_(2)_ = 12.69	0.002
Integrated/Balanced	17 (77.3%)	3 (20.0%)		
Not Integrated/Ambivalent	2 (9.1%)	2 (13.3%)		
Restricted/Disengaged	3 (13.6%)	10 (66.7%)		

Differences between the groups are reported as a result of statistical analysis. Concerning the seven dimensions of the IRMAG, significant differences were found with respect to richness of perceptions, openness to change, intensity of investment, coherence, and differentiation, where the pre-COVID-19 group reported higher scores than the COVID-19 group. In contrast, no significant differences were found with respect to social dependency and dominance of fantasies.

Moreover, comparing the maternal representation styles, significant differences emerged between the groups in the integrated/balanced (more prevalent in the pre-COVID-19 group) and restricted/disengaged (more prevalent in the COVID-19 group) representation styles.

### Interview Content of Women in The COVID-19 Group

The results of the interview content are reported below. Supplemental Material 2 reports quotations from specific areas of the interview for maternal representations during pregnancy. In the following section, the number of quotations is reported.

### The Desire for Motherhood in The Individual’s and Couple’s History

Some women reported that the desire for pregnancy had developed over time, while others had not developed this desire. In some cases, the pregnancy was planned according to social norms that expressed high social dependence leading to a merged desire for a baby (1.1). Sometimes, the COVID-19 pandemic seemed to influence the choice to become pregnant by suggesting a lack of awareness about motherhood (1.2). In other cases, the pandemic period, with the suspension of activities, led couples to discover a renewed closeness, increasing the women’s desire for motherhood (1.3).

### Emotional Reactions to Pregnancy (Individual, Couple and Family Emotions)

Sometimes uncertainty and a sense of temporal suspension due to the postponement of obstetric visits delayed the discovery and confirmation of pregnancy (2.3). However, when the pregnancy was confirmed, some women reported sharing joy and happiness with the partner (2.1), while others reported a more detached emotional reaction (2.2) or a sense of shock (2.4).

Some women reported regret at not having been able to share the announcement of their pregnancy with their relatives as they had hoped (2.5).

### Emotions and Changes During Pregnancy in the Woman’s and Couple’s Life and in Family Relationships

Most women reported a sense of discomfort and loneliness during pregnancy and anticipated distress regarding giving birth alone due to hospital restrictions on partner attendance. The real pregnancy appeared substantially different from what they had imagined, leading to sadness and disappointment due to the lack of participation, social recognition and sharing of the pregnancy (3.1; 3.2; 3.3).

Furthermore, the gap between the real and imagined pregnancy seemed to be increased by health care providers who tended to focus on physical gestation, neglecting the woman’s emotional experience (3.4). Participants reported the discomfort of not being able to share the pregnancy journey with other pregnant women (3.5). Some women, who were not totally able to acknowledge affective and physical changes, tended to express higher control over the pregnancy during the pandemic (3.6). Other women recognized a positive change in their relationship as a couple (3.7).

### A Woman’s Perceptions, Emotions and Fantasies About The “Internal Child”

The first perceptions of the baby occurred mainly through the ultrasound, which allowed the women to perceive the pregnancy as real. However, the COVID-19 restrictions limited partner attendance during visits and consequently their awareness about the internal child (4.1). As far as foetal movements were perceived, all the women recognized them as signs of the baby’s presence. However, some women interpreted foetal movement as the result of their own personal activity (4.2), while others perceived the internal child to have personal traits (4.3). In some cases, COVID-19 restrictions lead women to have a more intimate relationship with the foetus (4.4).

### A Woman’s Future Expectations About Herself as A Mother and her Child

Most women reported future expectations of themselves as mothers during the pandemic period. Sometimes, concerns about social isolation merged with the fear of being alone in providing care for the baby (5.4). Social isolation was sometimes positively perceived because it would allow the woman and the couple to develop a new routine as a family (5.5). Few fantasies merged about the child, and the baby’s image was mostly related to the parents’ features (5.1; 5.2; 5.3).

### A Woman’s Biographical Perspective Regarding her Present and Past Role as A Daughter

Some women had an image of themselves as a partially elaborated daughter, allowing an incomplete acquisition of maternal identity (6.1;6.2; 6.3;6.4). The COVID-19 restrictions sometimes seemed to represent an opportunity to develop disengagement from the role as a daughter (6.5).

## Discussion

The literature has shown that psychosocial risk factors can negatively impact the style of maternal mental representations during pregnancy by increasing the levels of restricted/disengaged and not-integrated/ambivalent representations and decreasing the levels of integrated representations (Ammaniti et al., 2013; Mascheroni et al., 2020). This study extended the literature showing that the COVID-19 pandemic increased the level of restricted mental representations and decreased the level of integrated representations in pregnant women with respect to the previous period (Agostini et al., 2009).

The high percentage of restricted/disengaged representations in the COVID-19 group could suggest that the pandemic increased women’s attention to concrete aspects of pregnancy to the detriment of emotional aspects. An analysis of the IRMAG dimensions and content may better clarify this point; in fact, the COVID-19 group reported lower levels of richness of perceptions, openness to change, intensity of investment, coherence, and differentiation than the pre-COVID-19 group.

The lower sensitivity to the emotional aspects of pregnancy in primiparas during the COVID-19 pandemic may be due to multiple merged factors from the content analysis, including greater consideration by health care providers of physical health instead of psychological health, a reduction in sharing the pregnancy journey with partners and family members and difficulty in celebrating pregnancy stages with others (e.g., ultrasounds) (Flaherty et al. 2022). The national and hospital restrictions due to the pandemic may have reduced the social recognition of future motherhood, increasing women’s difficulties in developing integrated maternal representations (Pajulo et al., 2001). However, other personal and relationship factors could moderate the impact of COVID-19 restrictions on the emotional experience of pregnancy. For instance, an affective marital relationship and an open attitude towards unexpected life circumstances (such as the COVID-19 pandemic) seem to promote couples’ shared experiences of pregnancy, increasing women’s emotional involvement. Thus, the pandemic restrictions could be perceived as an opportunity to facilitate a private space for couples, allowing the development of new pregnancy-related rituals and promoting the transition to parenthood (Imber-Black, 2002). In conclusion, on the one hand, our findings confirmed previous qualitative studies on the impact of the COVID-19 pandemic on the pregnancy experience (Flaherty et al. 2022); on the other hand, they allowed us to understand how the focus on concrete aspects may increase the risk for restricted mental representations compared to the pre-COVID period.

## Limitations

Despite its interest, this study presented some limitations. First, our sample size could be considered small compared to other studies conducted in the pre-COVID-19 period (Agostini et al., 2009; Ammaniti et al., 2013) due to the decision to include only primiparous women with low-risk pregnancies and without established psychiatric diagnoses. Moreover, several obstacles in recruitment and data collection during the COVID-19 pandemic negatively impacted the sample size. The restriction policies, the reduction of obstetric visits, and the reduction of women’s access to public hospital settings increased the difficulties in recruitment. Moreover, some individuals refused to participate due to the fear of contagion, while others could not participate due to testing positive for COVID-19 and/or being subjected to quarantine measures. We acknowledge that using in-person interviews may have reduced participation and that the use of online administration may have facilitated participation during the COVID-19 pandemic (Howlett, 2021). However, our decision was justified both by the necessity not to change setting in the pre-COVID-19 and COVID-19 periods and to overcome potential criticism of online administration, such as an interviewee’s reluctance to speak freely due to the presence of family members (Fry et al., 2021).

Furthermore, the fear of contagion, which lead some women to refuse participation, did not allow us to control social and clinical characteristics, and this may constitute a selection bias.

## Conclusions for Practice

This study appears to suggest some key points that could be improved for future pandemics. Pregnant women should be supported in the exploration of their inner world. It could be desirable to recommend the practice of keeping a pregnancy diary that could help women reflect on their own inner world in addition to identifying physical and relational changes. Second, it would be useful to provide online meeting groups under the supervision of a perinatal psychologist. This support group should help women increase their attention to their emotions, sensations and fantasies about themselves as mothers and their children. Providing a network of support from the early stages of pregnancy could increase social recognition of a future mother’s role, thus helping women in the transition to motherhood (Lee et al., 2019).

Finally, specific information on the pregnancy process and foetal development should also be provided through pregnancy-specific apps. This could allow women and their partners to be more aware of the pregnancy stages and their child’s development.

## Electronic Supplementary Material

Below is the link to the electronic supplementary material.

Supplementary Material 1

## Data Availability

Data are available by authors under reasonable request.

## Code Availability

IBM Statistical Package for Social Sciences (SPSS), for Macintosh, Version 24 (2017).

## References

[CR1] Agostini F, Monti F, Fagandini P, De Pascalis D, Sala LLL, Blickstein I (2009). Parental mental representations during late pregnancy and early parenthood following assisted reproductive technology. Journal of Perinatal Medicine.

[CR2] Ammaniti M, Candelori C, Pola M, Tambelli R (1995). Maternità e gravidanza. Studio delle rappresentazioni materne.

[CR3] Ammaniti, M., & Speranza, A. M. (1995). Rappresentazioni materne e attaccamento infantile disorganizzato. *Giornale di Neuropsichiatria dell’Età Evolutiva*.

[CR4] Ammaniti M, Speranza AM, Tambelli R, Odorisio F, Vismara L (2007). Sostegno alla genitorialità nelle madri a rischio: valutazione di un modello di assistenza domiciliare sullo sviluppo della prima infanzia. Infanzia e adolescenza.

[CR5] Ammaniti M, Tambelli R, Odorisio F (2013). Exploring maternal representations during pregnancy in normal and at-risk samples: the use of the interview of maternal representations during pregnancy. Infant mental health journal.

[CR6] Bowling, A. (2014). *Research methods in health: investigating health and health services*. McGraw-hill education (UK).

[CR7] Brooks SK, Weston D, Greenberg N (2020). Psychological impact of infectious disease outbreaks on pregnant women: rapid evidence review. Public health.

[CR8] Cast, A. D. (2004). Well-being and the transition to parenthood: An identity theory approach. Sociological perspectives, 47(1), 55–78

[CR10] Davis-Floyd R, Gutschow K, Schwartz DA (2020). Pregnancy, birth and the COVID-19 pandemic in the United States. Medical Anthropology.

[CR11] Delmastro M, Zamariola G (2020). Depressive symptoms in response to COVID-19 and lockdown: a cross-sectional study on the italian population. Scientific reports.

[CR12] Diamond RM, Brown KS, Miranda J (2020). Impact of COVID-19 on the Perinatal Period through a Biopsychosocial systemic Framework. Contemporary family therapy.

[CR13] Flaherty SJ, Delaney H, Matvienko-Sikar K, Smith V (2022). Maternity care during COVID-19: a qualitative evidence synthesis of women’s and maternity care providers’ views and experiences. BMC Pregnancy and Childbirth.

[CR14] Fry A, Mitchell SA, Wiener L (2021). Considerations for conducting and reporting digitally supported cognitive interviews with children and adults. Journal of Patient-Reported Outcomes.

[CR15] Hassanzadeh R, Abbas-Alizadeh F, Meedya S, Mohammad-Alizadeh-Charandabi S, Mirghafourvand M (2021). Primiparous women’s knowledge and satisfaction based on their attendance at childbirth preparation classes. Nursing Open.

[CR17] Howlett M (2021). Looking at the ‘field’ through a zoom lens: methodological reflections on conducting online research during a global pandemic. Qualitative Research.

[CR19] Imber-Black E (2002). Family rituals—from research to the consulting room and back again: comment on the special section. Journal of Family Psychology.

[CR20] Lebel C, MacKinnon A, Bagshawe M, Tomfohr-Madsen L, Giesbrecht G (2020). Elevated depression and anxiety symptoms among pregnant individuals during the COVID-19 pandemic. Journal of Affective Disorders.

[CR21] Lee K, Vasileiou K, Barnett J (2019). ‘Lonely within the mother’: an exploratory study of first-time mothers’ experiences of loneliness. Journal of health psychology.

[CR22] Mascheroni E, Faccio F, Bonassi L, Ionio C, Peccatori FA, Pisoni C (2020). Exploring differences in psychological aspects during pregnancy between cancer survivors and women without a history of cancer. Supportive Care in Cancer.

[CR23] Ostacoli L, Cosma S, Bevilacqua F, Berchialla P, Bovetti M, Carosso AR (2020). Psychosocial factors associated with postpartum psychological distress during the Covid-19 pandemic: a cross-sectional study. Bmc Pregnancy And Childbirth.

[CR24] Pajulo M, Savonlahti E, Sourander A, Piha J, Helenius H (2001). Prenatal maternal representations: mothers at psychosocial risk. Infant Mental Health Journal: Official Publication of The World Association for Infant Mental Health.

[CR25] Slade A, Cohen LJ, Sadler LS, Miller M (2009). The psychology and psychopathology of pregnancy. Handbook of infant mental health.

[CR26] Smorti, M., Benvenuti, P., Vanni, C., & Valoriani, V. (2010). Procreazione medicalmente assistita e rappresentazioni materne in gravidanza. *Procreazione medicalmente assistita e rappresentazioni materne in gravidanza*, 1000–1022.

[CR27] Smorti M, Ponti L, Ionio CA, Gallese M, Andreol A, Bonassi L (2021). Becoming a mother during the COVID-19 national lockdown in Italy: issues linked to the wellbeing of pregnant women. International Journal of Psychology.

[CR34] Smorti Martina, Gemignani Angelo, Bonassi Lucia, Mauri Giulia, Carducci Alessia, Ionio Chiara (2022). The impact of Covid-19 restrictions on depressive symptoms in low-risk and high-risk pregnant women: a cross-sectional study before and during pandemic. BMC Pregnancy and Childbirth.

[CR28] Stern, D. N. (1995). *The Motherhood Constellation: a unified view of parent-infant psychotherapy*. International Universities Press.

[CR29] Tambelli R, Odorisio F, Lucarelli L (2014). Prenatal and postnatal maternal representations in non-risk and at-risk parenting: exploring the influences on mother–infant feeding interactions. Infant Mental Health Journal.

[CR30] Tambelli, R., Odorisio, F., Trentini, C., & Ammaniti, M. (2008). Le rappresentazioni materne prima e dopo la nascita del bambino, in condizioni a rischio e non a rischio: indicatori predittivi della relazione madre-bambino nel primo anno di vita.Rivista di studi Familiari.

[CR31] Van Bussel JC, Spitz B, Demyttenaere K (2009). Anxiety in pregnant and postpartum women. An exploratory study of the role of maternal orientations. Journal of affective disorders.

[CR32] Wilson AN, Ravaldi C, Scoullar MJ, Vogel JP, Szabo RA, Fisher JR (2021). Caring for the carers: ensuring the provision of quality maternity care during a global pandemic. Women and birth.

[CR33] Zanardo V, Manghina V, Giliberti L, Vettore M, Severino L, Straface G (2020). Psychological impact of COVID-19 quarantine measures in northeastern Italy on mothers in the immediate postpartum period. International Journal of Gynecology & Obstetrics.

