# Supplementary material for: Prenatal Mental Representations in Italian First-Time Mothers Before and During the COVID-19 Pandemic: A Study with Interviews on Maternal Representations During Pregnancy
Source: Matern Child Health J. 2023 Jan 31;27(4):711–8. doi: 10.1007/s10995-022-03573-5 (PMC9888744; doi:10.1007/s10995-022-03573-5)
Supplement: Supplementary file 1 — Supplementary Material 1 [file 10995_2022_3573_MOESM1_ESM.docx]

Title: Prenatal mental representations in Italian first-time mothers before and during COVID-19: a study with Interviews for maternal representations during pregnancy

Journal name: Maternal and Child Health Journal

Authors: Martina Smorti *^ₐ^, Giulia Mauri ^ₐ^, Alessia Carducci ^ₐ^, Angelica Andreol ^b^, Lucia Bonassi ^b^

Corresponding author:

Martina Smorti, Department of Surgical, Medical and Molecular Pathology and Critical Care Medicine, University of Pisa, Via Savi 10, 56126 Pisa; Tel +39 050992370; fax +39 050993325; email: martina.smorti@unipi.it

**Supplementary material**

**Supplemental Material** **1 Analysis of content**

The content of each area was explored as follow:

*1) The desire for Motherhood in the personal and couple’s history*

- Pregnancy circumstance (i.e. pregnancy desired or undesired, planned or unplanned)
- Factors leading to pregnancy: objective and rational choices, social norms, personal and emotional aspects; desire for motherhood or desire for a baby
- Possible connections among these factors and COVID-19 pandemic

*2) Emotional reactions to pregnancy (personal, couple and family emotions)*

- Possible difficulties in recognizing/accepting the pregnancy
- Relation between these difficulties and COVID-10 restrictions (i.e. reduction, postponing obstetric visits, misinterpretation of pregnancy signs)
- Possible effects of COVID-19 pandemic on personal and couple emotions related to pregnancy (i.e. increased concerns due to COVID or joy despite COVID)
- Possible effect of COVID-19 on the mode and timing of announcement of pregnancy to family (i.e. on-line vs in-person announcement)

*3) Emotions and changes during pregnancy in the woman’s and couple’s life and in family relations*

- Physical, emotional, relational changes that have been mainly recognized in pregnancy during COVID-19 (i.e. increased emotional isolation or emotional connectedness during pregnancy in personal life, couple and family relations)
- Possible comparison between the real and imagined pregnancy: how COVID-19 pandemic (and related restrictions) move real pregnancy away from imagined ones

*4) Woman’s perceptions, emotions and fantasies about the “internal child”*

- More relevant aspects of internal child’s perception
- The role of COVID-19 restrictions on “subjective” experience of internal child

*5) Woman’s future expectations about herself as a mother and her child*

- The degree in which woman perceive herself in relation to her baby
- The measure in which these expectations are related to COVID-19

6) *The woman biographical perspective regarding her present and past daughter’s role*

- The impact of COVID-19 restriction on the role’s transition from daughter to mother
- How the reduced social relationship with own mother hindered the woman’s role transition from daughter to mother

**Supplemental Material 2.** The following table reports quotations from specific areas of Interview for Maternal Representations in Pregnancy with identification number (n) of women (W) who expressed (#Wn).

| 1.1  1.2  1.3 | **1. The desire for motherhood in the personal and couple’s history**  “*.. Well, we have been together for ten years, we got married two years ago (looks up) and after we bought a house and enjoyed a certain stability, it all started with the baby... I'm 38 years old so we couldn't wait any longer, even though I had tried to put it off as if I were still 15 years old” (#W1)*  *“…I am 34 years old, we had to get married but everything was canceled due to the COVID ... so both for the age and for the previous marriage decision…. we decided to anticipate the pregnancy” (#W3)*  *“…and it really happened, anyway let's say, in the months in which with work, I was in layoff, my partner instead was in smartworking, so let's say, we lived at home a lot in those months there...[…] Despite the context of COVID was negative I experienced it very well because we shared our domestic life ”(#W7)* |
| --- | --- |
| 2.1  *2.2*  2.3  2.4  2.5 | **2. Emotional reactions to pregnancy (personal, couple and family emotions)**  *“…I was very happy as was my partner... he didn't expect it, so we were all very happy, also our parents...” (#W3)*  *“…my partner started to cry he was very excited at the news of the pregnancy. I am a bit different because I was very happy, but I didn't feel strong emotions like him…[…]” (#W9)*  *“…I had to undergo a medical visit but, due to lockdown… all visits were postponed… although I imagined I was pregnant… we were living in a sort of limbo” (#W13)*  *“I had a delay but I believed it was because of stress. The COVID, my troubles on the work… I was really stressed …thus when I got pregnant, I was shocked … I was thinking “maybe the test is wrong!” … Day after day I began to accept understand the idea” (#W1)*  *“We were still in lockdown so we made a series of phone calls to announce it ... it felt very strange with mixed feelings of loneliness and happiness for the pregnancy” (#W2)* |
| 3.1  3.2  3.3  3.4  3.5  3.6  3.7 | **3. Emotions and changes during pregnancy in the woman’s and couple’s life and in family relations**  *“the only thing I regret in all this is that I would have liked to have lived through all the ultrasound of pregnancy together with my boyfriend” (#W4)*  *“This period I would have imagined totally different from how it went with the COVID... the visits in which only you can enter wearing a mask, the days after delivery...that not even your partner can come to visit you... we would never have imagined it...” (#W8)*  *“I've been alone a lot, it's a joy to have a baby but I thought it would have been more shared” (#W3)*  *".. I would have expected more sensitivity from the medical staff also and above all in a delicate period like the COVID one, I understand the stress and the overturning of the organization of the path of birth "(#W7)*  *“..the preparation course will be online, maybe it will provide information and will also be a comparison with regard to childbirth ... it is missing .. also comparisons with other pregnant women, even during the bi-test, I felt calmer because I could see other women, I could see the other bellies... I felt in tune with that environment...” (#W10)*  *“I haven't changed, I'm just more emotional, from a physical point of view nothing has changed, just my belly has grown… on the contrary, my in-laws have changed a lot and are very close to us” (#W11)*  *“So this lockdown maybe on the one hand it was also good... in a normal situation probably in these months my partner would have continued to travel, I would have been alone more often... I am glad to live the pregnancy with him day by day” (#W9)* |
| 4.1  4.2  4.3  4.4 | **4. Woman’s perceptions, emotions and fantasies about the “internal child”**  *“The most exciting part, which unfortunately my husband could not experience, was the very first ultrasound, because there I was able to realize I was pregnant” (#W5)*  *“Now that I am at home all day, it is calmer probably because I am less active... before when I went to work it was always on the move as mine is a dynamic job...” (#W6)*  *“I am used to hearing it a lot ... it listens to music, if it is very loud or with treble it starts to move a lot after being still.” (#W12)*  *I noted that the baby calm down when I embrace the belly… He (baby) likes when I touch him… I would never admitted my belly being touched as often happen to pregnant women… So the COVID helped me to avoid the others contact leaving me to exclusive contact with my baby” (#W11)* |
| 5.1  5.2  5.3  5.4  5.5 | **5. Woman’s future expectations about regarding herself as a mother and her child**  *“I imagine him with as much hair as my family has...” (#W3)*  *“... I have a hard time imagining my baby physically... I would like to be a conscious mother and not get anxious, but I don't know” (#W9)*  *“I hope our daughter looks like me... I hope I'm not too apprehensive a mother” (#W8)*  *“I repeat myself that I am not alone, that he (partner) is with me and will help me a lot. For instance, if the baby will cry maybe he would give me a break, … But, you know,… between COVID, the fact that we are alone, or I am alone, I am bit worry” (#W2)*  *“Immediately after the birth I believe that we will prefer to be among us, in the sense that we dedicate time, when possible, for the three of us” (#W4)* |
| 6.1  6.2  *6.3*  *6.4*  *6.5* | **6. The woman biographical perspective regarding her present and past daughter’s role**  *“I was a selfless child and I tried to obey even if there were no strict rules, but I have always been a responsible child probably because I had many younger siblings... " (#W3)*  *“I was always a very quiet little girl... and I probably think I was too quiet a child, almost boring” (#W1)*  *“They told me I was pretty quiet at least from what I know... the relationship with my parents was always good” (#W8)*  *“mmm, my parents tell me that as a child I didn’t let my parents sleep much but I had a good relationship with them and I still have today” (#W9)*  *“One time I visited my friend after the childbirth. At a moment arrived her mother. As she look her grandchildren for the first time she took the baby in her arms… I would never desire it … The COVID will allow me to cuddle the baby without this intrusion […] I have always perceived my mother so heavy” (#W12)* |
